# ‘It Feels Very Weird and Normal at the Same Time’: Sibling Perceptions of Their Relationships With an Autistic Brother or Sister With Complex Care Needs

**DOI:** 10.1111/jar.70009

**Published:** 2025-01-16

**Authors:** Louise Rixon, Richard P. Hastings, Hanna Kovshoff

**Affiliations:** ^1^ Centre for Research in Intellectual and Developmental Disabilities (CIDD) University of Warwick Coventry UK; ^2^ Intellectual Disabilities Research Institute (IDRIS) University of Birmingham Birmingham UK; ^3^ The Autism Community Research Network @Southampton [ACoRNS] and the Centre for Research in Mental Health, School of Psychology University of Southampton Southampton UK

**Keywords:** autism, complex care needs, interviews, reflexive thematic analysis, sibling relationships, siblings

## Abstract

**Background:**

The impact of having a disabled brother or sister on siblings' psychological well‐being and sibling relationships has been the subject of several research studies. However, research which focuses on the relationship between siblings and their autistic brother or sister with an intellectual disability and complex care needs is rare. We explored siblings' views and experiences of their sibling relationship with their autistic brother or sister with complex care needs.

**Method:**

Eleven children and early adolescents (4 male/7 female) between the ages of 8 and 14 years took part in semi‐structured interviews with questions focussing on their relationship with their autistic brother or sister who had complex care needs. Reflexive thematic analysis was used as a guide to analyse the data.

**Results:**

Four themes are presented: positive interactions bring joy, sibling conflict is driven by verbal interactions, behaviours may have different meanings for the sibling, perceptions of change in the sibling relationship.

**Conclusions:**

The siblings of autistic brothers and sisters with an intellectual disability and complex care needs described warmth and positivity. Siblings of autistic children, who have complex care needs, deeply valued their interactions with their brother or sister despite reciprocity being on their terms. When autistic brothers and sisters had some functional spoken language, this often changed the context for the siblings relationship; perhaps leading to an increased chance of conflict or perceived negative experiences. These findings highlight the importance of understanding the individual meaning of these sibling relationships.

## 1 Introduction

The sibling relationship is often one of the most enduring relationships across the lifespan, and is an important context for children and young people's development (Douglas, Bagawan, and West 2023). This meaningful relationship develops at birth and extends through the lifetime to death and influences many aspects of the individuals lives such as well‐being, development and psychosocial functioning (Rum et al. 2024). Research aiming to understand these relationships when one sibling is autistic has been the focus of several studies across the last 20 years (e.g., Trew 2024; Burnham Riosa et al. 2023; Guidotti et al. 2020; McHale, Updegraff, and Feinberg 2016). However, there remains an inconsistency in the literature (Leedham, Thompson, and Freeth 2020) with conflicting findings on the outcomes of these siblings, with a risk of adverse developmental outcomes and adjustment issues reported in studies (e.g., Park et al. 2023; Kaminsky and Dewey 2001) alongside reports of positive outcomes such as siblings being more likely to exhibit higher empathic and pro‐social behaviours (Orm et al. 2022; Shivers, Jackson, and McGregor 2019; Mascha and Boucher 2006). Siblings may have increased caring responsibilities (Pavlopoulou and Dimitriou 2019), they may feel embarrassment or a sense of burden in their future responsibilities (Wintgens and Hayez 2003). These relationships can also be characterised by feelings of warmth and affection (Guidotti et al. 2020), decreased conflict and increased resilience (Schmeer et al. 2021) and may result in increased pro‐social and empathic behaviours in the sibling (Orm et al. 2022; Mascha and Boucher 2006).

Watson, Hanna, and Jones (2021) reviewed research on the experiences of being a sibling with an autistic brother or sister with a wide range of autism symptoms and care needs, across 15 qualitative research studies. The age in the review studies ranged from 5 to 29 years (1 study, 14 siblings); 12–20 years (1 study, 14 siblings); the remainder being within the 7–18 age range. None of these studies explicitly reported the focus of the sibling samples being those whose brothers or sisters might represent a complex care needs profile. Four analytical themes were identified in the meta‐synthesis: (i) impact on self and personal development; (ii) interaction with their autistic sibling; (iii) interaction with others and (iv) experiences of coping. Whilst there were some challenges, such as coping strategies and their interaction with others, there were also positive experiences reported across the sibling relationships. These included feelings of pride, appreciation of the uniqueness of their brother or sister, and the positive impact on their own personal attributes, for example an increased empathy and understanding and feeling good about their ability to help out.

Whilst there continues to be ample research focussing on these sibling relationships, less have focused on the sibling relationship when one child is autistic and has an intellectual disability and complex care needs. Researchers have identified, using cluster and latent class analysis approaches, meaningful sub‐groups of autistic children drawing from multiple indicators of needs. For example, Rixon et al. (2021) identified five needs‐based clusters/sub‐groups of autistic children including 26% in what the researchers labelled as a “complex needs” group (more severe autistic characteristics, significant adaptive skills limitations, and relatively high levels of behavioural and emotional problems). In a partial replication study focusing on autistic sub‐groups in a population of children with intellectual disability and autism, Rixon, Hastings, and Kovshoff (2022) used latent profile analysis to identify sub‐groups of autistic children with intellectual disability, again identifying a complex needs group. Rosello et al. (2021), utilising cluster analysis, identified three clusters including 40% in a ‘high severity’ group, (presenting high symptom severity on the DSM‐5 criteria and the Social Communication Questionnaire).

In a recent review of the (qualitative and quantitative) literature on siblings of individuals with severe intellectual and developmental disabilities (Roberts 2021) 27% of included studies researched sibling populations where their brother or sister was also identified as autistic (4 autism studies age ranged from 3 to 11 years; 6–18 years; 11–17 years; 11–18 years; 18 years+). The type of disability, in addition to severe intellectual or developmental disabilities, was an important influence on research findings in the review. For example, siblings of autistic individuals were more pessimistic about their sibling's future, and felt their relationship had been adversely impacted, when compared with siblings of those brothers and sisters with Down syndrome (e.g., Orsmond and Seltzer 2007). Roberts (2021) also concluded that the experiences of siblings of individuals with severe intellectual and developmental disabilities were not well represented in other recent reviews of the experiences of siblings of individuals with developmental disabilities, suggesting that additional research on this group needs to be more prominent in future.

Given the lack of attention to siblings' experiences of the sibling relationship in the context of autism and complex care needs, the main aim of the present research was to understand this relationship when one sibling is autistic, with an intellectual disability and, specifically, has complex care needs. We conducted semi‐structured interviews with the siblings to explore their views and feelings about the nuances of their relationship. Whilst there has been an increase in the studies that evaluate sibling relationships from the siblings' perspectives, there is still some reliance on the parental voice to gain insights, relying solely on parental perceptions may not provide a clear understanding of this relationship (Braconnier et al. 2018). Within this specific population, the siblings of an autistic brother or sister with complex care needs, speaking to the participants directly highlights the importance of hearing the voices of this niche cohort of siblings. To further understand how autism and complex care needs are perceived within sibling relationships by the siblings themselves.

## 2 Method

### 2.1 Methodological Approach

This qualitative study was conducted using the principles of Braun et al. (2022) reflexive thematic analysis framework (Table 1). Data were gathered through one‐to‐one semi‐structured interviews, developed and delivered by the first author, in consultation with the other authors. This methodological approach to data collection was utilised for its flexibility in providing the researcher with the means to gather in‐depth accounts of people's experiences whilst exploring subjective viewpoints. The semi‐structured interview approach offered an accessible method to interpret and explore the interview dataset. The use of an interview schedule provided some structure to the interview, particularly pertinent when interviewing children, whilst giving the respondent the freedom to respond with their own thoughts and viewpoints (Appendix A). Consisting of six key phases (Braun et al. 2022), reflexive thematic analysis allowed the researcher to determine the outcome and focus rather than being bound to a specific theoretical framework, developing, analysing and interpreting patterns across the qualitative dataset.

**TABLE 1:** Participant information (sibling) and characteristics of their autistic brother/sister.

Participant number^a^	Participant gender	Participant age (*m* = 10.36; SD)	Autistic sibling	Autistic sibling age (*m* = 11.36; SD)	Diagnosis, communication needs^a^
001	Male	12	Male	6	Autism, non‐verbal
002	Female	11	Male	11	Autism, non‐verbal
003	Female	8	Male	9	Autism, non‐verbal
004	Female	9	Female	16	Autism, verbal, cerebral palsy
005	Female	8	Female	15	Autism, limited language
006	Female	10	Male	15	Autism, non‐verbal
007	Female	12	Male	11	Autism, non‐verbal
008	Male	10	Male	5	Autism, non‐verbal
009	Female	14	Male	16	Autism, limited language
010	Female	9	Male	14	Autism, verbal
011	Male	11	Male	7	Autism, non‐verbal

^a^ As described by the primary parent carer.

The stages of reflexive thematic analysis used within this study consisted of:

1. Familiarisation—the researcher listened to the audio files, transcribed and then repeated the process to re‐read and check the audio to transcription were accurate. Notes were taken within this process to record any initial thoughts and findings.
2. Coding—the researcher created a new coding book in NVivo software, recording each coding decision, this process was then repeated manually and the two coding processes were combined, checking that all data relevant to the research question had been tagged. The researcher felt that by utilising both software and manual processes gave the most in‐depth understanding of the transcripts (rather than relying on software alone).
3. Initial theme generation—a process of clustering and then re‐clustering the codes to explore potential patterns of shared meaning. Each code was reviewed individually using a manual clustering process, thus providing a more comprehensive visual overview of the clusters.
4. Reviewing and developing themes—the researcher then reviewed and shared the initial themes with the co‐authors, continuing a process of reworking, refining and reviewing themes.
5. Refining, defining and naming themes—after a process of further refining and discussion, short theme definitions were written to capture the scope and core concept of each theme. Further discussion and refinement ensured the themes captured meaningful patterns, considering the story each theme told and the overall analytic story.
6. Producing the report—the researcher bought together the data extracts and analytical commentary to tell the story of each theme, contextualising the overall analytical story in relation to their existing knowledge.

The first author/interviewer's background and knowledge of autism were discussed by the research team prior to commencing the study. As a parent of an autistic child with significant and complex care needs, who also has two siblings, the interviewer had not only first‐hand lived experience of autism but also of family life within these parameters. Although not the sibling of an autistic person herself, this lived experience perspective provided the interviewer with rapport and trust with the parent carers and the siblings at the initial pre‐interview meetings. Being an ‘insider researcher’ (Braun et al. 2022) can enhance access and recruitment to the target group, built rapport and trust, and provide more depth to the design and analysis, and, in turn shape the findings and conclusions of the study (Kacen and Chaitin 2006). The change in the nature of the ‘researcher/researched’ relationship can affect the information the participants are willing to share (Berger 2015), making the both the parent carer and the siblings more comfortable in sharing very personal details of their lives. Within the current study, providing the siblings with personal information pertaining to the interviewers' home life was designed to obtain more in‐depth discussions.

### 2.2 Participants

Participants were 11 siblings (Mean age = 10.36 years, SD = 1.77) of autistic children with significant and complex care needs (Mean age = 11.36 years, SD = 3.93). Study inclusion criteria for the siblings invited for the research were that they were: (a) neurotypical, (b) within the age range of 8–15 years 11 months, and (c) living in the same house as the autistic child. Inclusion criteria for the autistic children (the brother/sister) were that they were within the age range of 5–17 years 11 months and had complex care needs, operationalised as: (a) attendance at a specialist school or additional learning needs unit within mainstream school; and (b) a diagnosis of autism, with low adaptive skills, restricted social communication skills and limited verbal communication (as reported by the parent carer). Limited verbal communication was specified as being, for example, echolalic, repetitive with minimal understanding or minimal words with limited understanding, to the parents. The study exclusion criteria were: (a) that the sibling had a diagnosed cognitive disability and (b) that the sibling did not provide assent to participate in the study.

Key participant characteristics are presented in Table 1. Of the siblings, three were male and eight were female, four were the older sibling, six the younger sibling and one twin (See Table 1). Within this study, one sibling per family was included. Within families of multiple siblings the final decision for inclusion was decided by the parent, with assent from the child. Fourteen parents initially expressed an interest in their child taking part in the study. Of these, two children chose not to take part and the third had a birthday which then resulted in him being above the upper age limit for the study. Giving the siblings agency to make their own decisions within this research was important, and it was highlighted throughout the pre‐visit and interview stages that the final decision of whether to continue was the child's and not the parents'.

### 2.3 Procedure

Full ethical approval to conduct this research was granted through the first author's institution. The parents of prospective sibling participants self‐selected by responding to an online flyer placed on the social media pages of a parent carer peer‐to‐peer support network and an autism family support service. The parents were then contacted via telephone to explain the purpose of the study, an information sheet for both parents and siblings had been emailed prior to this contact. This gave the parents the opportunity to discuss and decide whether they felt their autistic child met the inclusion criteria including, ‘complex care needs’.

The parental interpretation of the inclusion criteria of ‘autistic with complex care needs’, and how the parents understood, described, and communicated the individualities of their autistic child's disability. provided an opportunity for the parents to interpret what they felt was ‘complex’ within the care provided for the individual autistic child or young person.

The majority of the siblings who participated, nine of 11 children, had an autistic brother or sister who exhibited very limited or no verbal language ability and were described as needing a high level of support by their parents. However, within the autistic group were two teenagers, who were identified as having a higher verbal ability than the rest of the cohort but were still described, by their parents, as needing a high level of care. It was decided to continue with the interviews and to include their siblings within the study, thus providing an opportunity to explore how verbal language ability, or the challenges of communicating verbally, impacted the sibling relationship within the study.

A pre‐interview meeting was arranged for the parents and the siblings who were eligible and had agreed to participate in the study. During the pre‐interview meeting, the siblings were informed of the voluntary nature of their participation, the use of the data, and the dissemination of the data once analysed. After receiving the verbal assent of the siblings, and after an agreed time period to allow for reflection of their decision to participate, the parents were asked to sign the consent form prior to interview dates being agreed.

Eleven children, whose primary parent carer had identified them as being the sibling of an autistic sibling who had and significant and complex care needs, were interviewed between April 2022 and June 2022. Interview questions were initially designed by the first author and discussed and revised by all three authors, Appendix A (interview protocol). The first question, ‘tell me about who lives in your home’, was designed to begin conversation and to put the participant at ease, following with open questions to gather information about the siblings' thoughts and feelings about their relationship with their brother/sister with autism.

Interviews were carried out in the sibling's homes with the primary parent carer in an adjoining room. One interview was carried out in a garden with the parent within sight but not within earshot. The siblings were asked to confirm that they were comfortable with this arrangement before the interview commenced. Due to the potentially sensitive nature of the questions, the researcher reminded the siblings that they could stop or pause the interview at any point or ask for their parent to be present (only one child requested a parent remain in the room). The interviews followed a semi‐structured format with a focus on the siblings thoughts and feelings of their relationship with their autistic brother or sister.

On completion of the first two interviews all three authors reviewed the sibling responses to the questions and an additional question was included for the remaining participants to focus on experiences of family holidays. The interviews were audio recorded and transcribed verbatim into written text. Interviews were checked for accuracy against the audio tapes before being deleted. Field notes were taken during, and additional notes made immediately after, each interview to capture any immediate thoughts and observations.

### 2.4 Coding and Analysis

Each interview was transcribed within 24 h of the meeting taking places with the siblings, with interviews ranging from 8.14 to 25.01 min, with an average time of 14.56 min. This procedure allowed for any relevant, and newly identified, additional questions to be included in further interviews. For example, after the first two interviewees discussed family holidays, all following interviews included a question about this subject.

Each of the individual interview recordings were reviewed three times by the first author to allow for familiarisation of the content, to provide initial ideas for coding, and to ensure the accuracy of the transcription. On completion of the verbatim transcription of the interviews, the data were analysed using an inductive, data‐driven approach. The initial coding stage was a recursive process, data were examined, codes identified, and then repeated, this provided a large number of codes, numbering 44 in total. Initial coding was conducted using NVivo software to organise and store the interview transcripts (Lumivero 2020). To further explore these codes, similarly coded extracts along with a description of each code were manually inputted into a spreadsheet. This resulted in 21 codes which were refined and combined, through discussion and review with all authors, into six themes and 15 sub‐themes. Further discussion, within the research team in regular meetings, combined the codes into four themes (Table 2).

**TABLE 2:** Themes.

Theme	Name	Description
1	Positive interactions bring joy	The interactions between the siblings—shared experiences and connections
2	Does verbal interaction increase sibling conflict?	When the autistic child has language is there a change in the levels of conflict described by the sibling?
3	Behaviours may have different meanings	How the behaviours of the autistic children are understood by their siblings and how these may be confused or contradictory in their meaning
4	Change within the sibling relationship	How the siblings felt their relationship had changed in terms of changes over time and changes they felt they could make

The field notes, taken during and following each interview, were included throughout the analysis of the interview data. These notes provided an additional layer of descriptive analysis and contextual information, including how participants reacted to questions, the environmental context, behaviours, and non‐verbal cues that may not have been fully captured through the audio recordings.

## 3 Findings

The siblings, in childhood and early adolescence, provided an insightful understanding of the significant and complex care needs of their autistic brother or sister and how this impacted the sibling bond. Findings reflected both the nuance of the siblings' individual relationships and the similarities in experience of having an autistic brother or sister with significant and complex care needs. The siblings displayed an openness when discussing their relationships and the joy many felt when interacting with their autistic brother or sister. However, not all of the children had a close sibling relationship, and some siblings described a level of conflict that existed between them. There was an emphasis on the importance of friends away from the home and the recognition that, for some children, time away from their autistic sibling was necessary to give them respite from their home environment and their sibling's care needs and behaviours.

### 3.1 Theme 1: Positive Interactions Bring Joy

The siblings discussed different elements of their relationship with their autistic sibling, their interaction, shared experiences and the connection between them.

When asked to describe their autistic brother or sister, the responses from the siblings were mostly positive. Words commonly used were, funny, happy, loving, cheeky, unique, and generous but also, weird and different. Many of them described enjoyable aspects of their relationship with their autistic brother or sister including shared experiences and playful interactions. These stories were told with love and positive affect—siblings spoke with smiles and a palpable sense of joy in sharing details of their relationship with their sibling. This joy was particularly evident when siblings shared details of their siblings with the most complex care needs. For example, sibling participant 6, a 10‐year‐old girl, described an interaction with her non‐verbal older brother which demonstrated the joy of a connection between them:

But when he goes to the park, he mostly loves climbing. He loves it when I try the same thing as him. Once I was really scared, I was really scared, and B was cheering on me to get up, which was nice and I was really proud that he did that’ (sibling participant 6).

The siblings were eager to talk about the positive elements of their relationship, seemingly wanting to communicate the close connection between them and their sibling and how this made them feel. ‘she's fun and she's funny’ (Participant 5). When asked what it is like living in the same house as his non‐verbal brother, sibling participant 8 replied, ‘Fun! I love it’. Sibling participant 3 described her non‐verbal brother as, ‘he plays with me a lot… cause he's friendly, he plays with all of us’ and sibling participant 2 discussed how her twin brother made her feel as, ‘makes me to feel happy it does, I quite love him a lot (he) makes me happy’.

They shared many close moments with their autistic brother or sister, and these interactions were lovingly participated in, even though they were often not what the sibling would have chosen to do, as explained by sibling participant 11:

Like recently he's been wanting to play floor is lava. It's were you say ‘floor is lava’ and then ‘5,4,3,2,1’ and you have to get on something. I don't enjoy the game but I like doing it with him.

Often interactions between the siblings and their autistic brother or sister were described as a two‐part experience. First, happiness often came from the sibling instigating the activity and receiving positive feedback from their autistic brother or sister. Second, if their autistic brother or sister executed a task or activity that made the sibling feel happiness, regardless of whether they had instigated the interaction or not. siblings described how they played with their autistic brother or sister and appeared to both enjoy the interaction and the time they were spending with them, regardless of whether the play was reciprocal.

I love it because, he likes playing with all of us and he likes playing with balls and I pass him the ball and sometimes he throws it back or rolls it or does that and sometimes it goes behind him (sibling participant 8).

A lack of reciprocal play was seen between many of the siblings and their brother or sisters, as recounted by sibling participant 2, ‘I'll line his cars up and then he'll play with his cars’. This was seen again between sibling participant 8 and his brother, ‘he plays with them but not with me’, and sibling participant 5 and her sister, ‘(we) play sometimes, together but not together’. Whilst these interactions were expressed as playing *with* their brother or sister, the actual activity described by the sibling was of them providing the *means* for the autistic child to embark on a solo play activity. However, the siblings were often enjoying this as much as their brother or sister.

Whilst, at times, they described relationships that could be misunderstood by those external to the siblings as one sided, it was evident that even the smallest of positive shared interactions made them happy. Often the ‘fun’ described was when their autistic brother or sister responded with a positive reaction to a simple gesture, such as a clap or a ‘high‐five’. Engagement was welcomed regardless of the complexity of the involvement behind it, for example sibling participant 7 described a game she played with her younger non‐autistic sister. The children used their autistic brothers' mattress topper as a slide from the bed to the floor, and while their brother would not join in the activity, their enjoyment came from his positive reaction watching their game. The simplest reaction, to an action the siblings had instigated, was providing fulfilling and joyful moments in their relationship.

### 3.2 Theme 2: Does Verbal Interaction Increase Sibling Conflict?

When asked what the difficult parts of their relationship were, the siblings mainly responded that there were not any, with only the two siblings of the children with some spoken language responding that there were. The siblings of the children with no, or very limited language, (e.g., echolalic, repetitive with minimal understanding or minimal words with limited understanding), were often reticent to say anything overtly negative, keeping their descriptions mainly to behavioural descriptions that could be perceived as out of the control of their brother or sister, such as ‘meltdown’. These siblings used more negative language when describing their autistic brother or sister's behaviours rather than negatives in their relationship with them.

However, these siblings, with a non‐verbal brother or sister, or with very limited language, (e.g., echolalic, repetitive with minimal understanding or minimal words with limited understanding as reported by the parent), reported that their relationships were, in the main, non‐combative. However, with gentle probing, it became evident that the reasoning behind these responses was not always about a lack of conflict, but a lack of verbal language in which these antagonistic exchanges could occur. Therefore, the lack of verbal communication from the non‐verbal autistic children may have equated to a perception of absence of conflict in the relationship to the siblings. This was expressed by sibling participant 1, ‘He can't talk or anything so he's not going to annoy me by doing anything really’, sibling participant 6, ‘We can't really fight as he has no words to say’ and sibling participant 8, ‘he doesn't understand, so I never fight with him, he doesn't understand anything because he's autistic’.

When autistic children used spoken language as their primary means of communication, the siblings were more likely to describe conflict as being prevalent in their relationship. The two verbal autistic teenagers were the only two who were described by their siblings as actively provoking them (sibling participants 4 and 10), invoking annoyance through verbal conflict. Both siblings were the younger, pre‐teen, sisters of a teenage older autistic brother or sister and were described by their sisters as being, ‘annoying’, ‘mean’, and ‘grumpy’. These were also the only two siblings to share how they used their bedrooms as a sanctuary to escape from the behaviours and unwanted attention of their brother or sister. They expressed how they needed time away from their brother or sister, recognising that they required their own space for their own mental wellbeing, ‘so basically my room is my shield’ (sibling participant 10). Sibling participant 10 also discussed how holidays involved travelling in separate cars and staying in separate rooms as, ‘there's no way to separate each other when we're there, it's a bit of a problem’. She spoke of her autistic brother being able to dictate what would happen, for example wanting time on the trampoline or the computer, and having to immediately stop and hand over to him.

Conflict also appeared to permeate many of the aspects of the interactions between sibling participant 4 and her autistic sister, often creating an atmosphere where she needed to mentally and physically remove herself from the environment. She spoke of the conflict as something *she* was responsible for, as she ‘bothered’ her sister. She described feeling isolated and feeling unable to talk to friends about the problems in her life, using her room and online games to provide a barrier between herself and the issues within their relationship and her sister's behaviours.

I sometimes just go in a different room or I sit through it or I can just go on my Switch and play with my friends. Because I do talk to my friends and sometimes, I have the door shut and I put my headphones on and I talk to them but I don't tell them about it or anything.

She also felt that school was often a catalyst for her sisters' behaviour; ‘she's quite mean sometimes, especially when she comes home from school’, but lacked the understanding of why school was creating problems for her autistic sister. She did, however, acknowledge that her sister behaved in a more positive manner when in the company of other people. It may well be the case that maintaining positive behaviours at school was creating pressure for her sister, that was then subsequently released as soon as she returned to their home, with her sibling as the target for her negative behaviours.

Across all of the siblings, sibling participant 4 was the only child who did not describe any positives within the relationship. The interaction between the sisters was minimal and was limited to brief online episodes, playing Roblox or Fortnite (online gaming platform and game creation system) together. The sibling felt this was easier as, ‘we don't fight as much’, when they played within an online medium. This description of online gaming together was also evident in the relationship between sibling participant 10 and her verbal brother, ‘we can see each other and play together but separately’. These two siblings were able to manage their relationships with their autistic brother and sister by providing a medium where there were no verbal interactions and they were able to play together but in separate rooms.

### 3.3 Theme 3: Behaviours May Have Different Meanings

The siblings discussed many behavioural aspects of their relationship with their autistic brother or sister. The meanings behind these behaviours were not as simple as being ‘good’ or ‘bad’, but instead where often described in contradictory terms, for example a pinch meaning a positive interaction:

When he pinches people, it means he's happy (sibling participant 8).

In all but two of the siblings, they did not see the biting, pushing, smacking and pinching as being harmful, instead they were conceptualised as merely an intrinsic part of their brother or sisters' nature. For example:

Sometimes when I walk past him, he just hits me…I don't really care (sibling participant 11).

Sibling participant 2 spoke positively about her non‐verbal brother's meltdowns as being a time when he would seek her out for comfort, putting his head against hers, a rare, treasured moment of acknowledgment in a relationship that often, lacked positive physical contact. Within this relationship, the sibling discussed how she longed for her brother to want to be more involved with her, to seek her out for comfort rather than her mother.

Descriptions of harmonious sibling interactions were often intertwined within a stressful environment, producing blurred boundaries between positive and negative experiences, as described by sibling participant 3:

So, it's like living with a noisy person who always screams when you wake up in the morning and whenever you try to play with him when he's in a bad mood he'll probably kill you (laughs) cause he has to be in the right mood, but I love playing with him, he's fun.

While the siblings mostly valued their time with their autistic brothers and sisters, and spoke positively of their relationship, they often described experiences that were blurring the boundaries of what may be considered ‘normal’ sibling interactions. For example, one sibling participant (6) described her teenage brothers' behaviours as ‘so funny’, even though these included climbing out of her bedroom window onto the roof and blocking the toilets with paper. In many instances the siblings were not acknowledging the physical elements of their relationship as being unusual.

The siblings described how their interactions with their autistic brothers and sisters, were shaping the relationships they had with peers outside of the family home. They described that they wanted their friendships with peers to be kept away from their home, with few taking friends home for meals or activities, preferring to keep a distinct separation between their home and social/school lives.

Not normally because of T, I normally go to theirs instead of them coming to ours, normally (sibling participant 10).

And do your friends come to your house? Not really, I mostly go to them (sibling participant 7).

Whilst the siblings mostly confirmed that their friends knew that they had an autistic brother or sister with complex needs, only one wished that they attended the same school. The older siblings described time away from the home and time spent with friends as more important to them. Younger siblings described how time was spent within the family home with their autistic brother or sister and less time spent with friends.

We don't go out, we stay here and play, she hates shopping and things (sibling participant 5).

Sibling participant 10 described how she only told very close friends about her brother:

Not really, like I told them about it but then it's not that sort of thing that I would just tell them that he's autistic, I wouldn't just tell them. Some ones that I could 100% trust, then yes which is E–, E– and G–, they're the only ones that I've told but then everyone else not 100%.

Siblings also described how they kept a distance between their friends and their brother or sister.

No he's just in the background, he's down here and I'm upstairs. My friends don't really think that much, he's just around (sibling participant 9).

I've got one of my friends he thinks she's quite annoying as well. And the other ones I don't know really what cause I haven't really told them about her. Most of them they don't know her (sibling participant 4).

### 3.4 Theme 4: Change Within the Sibling Relationship

Change within the relationship was discussed openly by the siblings, both in the context of changes over time (growing up) and the changes both they and their autistic brother or sister could make individually to have a more positive relationship with each other. Change was recurrent throughout the interviews, meaning many things to the siblings, here it is discussed under the broad banner of change within their relationship and what it meant to the individual children.

The siblings recognised that there had been changes in their relationship as both they, and their brother or sister, had grown older. They also recognised their role as a sibling had often changed too, moving from a peer to a role with more caring responsibility, and the recognition that whilst they and their brother or sister had aged, they had not developed at a comparable rate to them. In addition, some siblings described that as their autistic brother or sister with complex care needs grew older, that externalised behaviours were often replaced with more internalised behaviours,

Back then he just used to cry and now he doesn't cry, he just hits and bites people. He just doesn't know what to do, he gets frustrated (sibling participant 11).

It was also common for the younger siblings to feel ‘older’ than their autistic brother or sister, regardless of their age, due to caring responsibilities, or ‘looking after him as a big sister’ as described by sibling participant 6. Siblings also described how they felt they could change to improve their relationship with their brother or sister. They appeared to understand that whilst their autistic brother or sister dominated the narrative of their relationship, there were, perhaps, some adjustments they could make to improve their sibling connection. One sibling described how he felt he could change:

(I could) play with him more and talk to him more and so he can learn more stuff. I just want to be a better brother (sibling participant 8).

Siblings also recognised that they also wanted their brother or sister to change for them to have a better relationship with them. Having the opportunity to spend more time with their autistic brother or sister outside of the family home and managing some of their more difficult (for the sibling) behaviours were discussed. Whilst most of the siblings maintained that they were happy in the relationship, there were many things they wished they could change about their brother or sister including, decreasing anti‐social noises (such as screaming); normalising eating habits and a reduction in dangerous behaviours, such as running away.

## 4 Discussion

The present study sought to understand the relationship between non‐autistic siblings of autistic brothers and sisters, with an intellectual disability and complex care needs, from the non‐autistic siblings perspective. Four themes arose from the interviews with the siblings: (1) positive interactions brings joy; (2) does verbal interaction increase sibling conflict; (3) behaviours may have different meanings; (4) change within the sibling relationship. Findings suggest positive affect between siblings especially salient in the relationship descriptions of an autistic brother or sister with limited verbal or non‐verbal communication. In line with existing autism sibling research, siblings in the present research described a strong emotional bond, and warmth and affection for their autistic brother or sister (e.g., Guidotti et al. 2020; Iannuzzi et al. 2021). Our findings also corroborate existing research in that siblings of autistic children were eager to interact with their autistic brother or sister with complex needs. Even the smallest interactions were mentioned as bringing joy to the sibling of an autistic child regardless of whether the sibling was interested in the chosen activity. It was clear that a shared interaction was important for the relationship not the shared interest in the activity itself.

Thus, contrary to some previous research (e.g., Angell, Meadan, and Stoner 2012; Tomeny et al. 2016; Walton 2016), the siblings in the current study were mostly not reporting a poorer sibling relationship or increased feelings of frustration and stress when they had an autistic brother or sister with complex care needs. The two siblings who reported the least warmth, and the highest levels of conflict in the sibling relationship, were those who had an autistic brother or sister with both complex care needs alongside good levels of spoken language and communication.

The need to distance themselves from their autistic brother or sister is in line with past literature which highlights the desire to remove themselves from the situation as being self‐protection (Guidotti et al. 2020; Orsmond, Kuo, and Seltzer 2009). This may also explain the use of electronic gaming to provide a virtual barrier between the sibling and their autistic brother or sister.

The siblings spoke of the interactions between themselves and their autistic brother or sister as different, but not as difficult, echoing past research findings (Bachraz and Grace 2009). Behaviours such as meltdowns, dangerous actions (e.g., climbing out of an upstairs window), and physical interactions (e.g., accepting that pinching someone to show happiness was a normal behaviour) were characterised as a normal part of their lives and their sibling relationship with their autistic brother or sister. This raises an important consideration for how interactions are judged by people sitting outside of the sibling relationship (e.g., parents/researchers/clinicians). For example, previous research has often characterised these shared interactions as being of ‘poorer’ quality, due to the autistic child not directly reciprocating, or reciprocating in a way that an outsider to that relationship may not fully comprehend (e.g., Zucker et al. 2022). It was clear from the findings of this study that even when the autistic children engaged in stereotypically ‘negative’ behaviours (e.g., pinching or hitting), these were not always perceived negatively or antagonistically by the siblings. Instead, the siblings described deriving enjoyment from receiving feedback from their autistic brother or sister, and especially from the time they were spending with them. It was the autistic children described (by their parents) as having the highest severity of autism symptoms and complex care needs, who had the warmest relationships with their siblings; and these children were mostly those with either very limited or no spoken language. Of course it must be considered that the siblings with the brothers and sisters with the most complex care may not always vocalise their true feelings, perhaps feeling guilt at discussing the negative aspects of a relationship with their autistic brother or sister. This may be particularly pertinent if the siblings were reminded that they should care for their brother or sister because of their autism. The use of an ‘insider researcher’ could also exacerbate this, particularly if already known to the family, but could also provide a level of comfort and understanding that also increases the depth of information given. The potential loss of objectivity being weighed against the established intimacy and knowledge (Unluer 2012).

This study suggests that the siblings felt they were the older sibling, regardless of whether they were actually older or younger. However, unlike Corsano et al. (2017), the siblings did not voice concerns about future caring roles but were observing that, whilst they had grown older, both physically and mentally, their autistic brother or sister had not. Many of the siblings reported concerns that they were not doing enough for their autistic brother or sister, with complex care needs, often wanting to improve themselves to improve the sibling relationship. These observations suggest that the siblings are not necessarily thinking about their future responsibilities as described in other research (e.g., Iannuzzi et al. 2021; Petalas et al. 2012) but were concerned with their relationship at the current time, and how it could be improved. However, it is possible that the lower age range of the siblings interviewed (8–14 years), may have meant that they were not at an age when they were able to perceive what their future lives would entail.

Consistently, the siblings were very present in their relationships, with an awareness of the needs of their autistic brother or sister at that time. Siblings were aware of their autistic brother or sisters' needs both in and out of the family home, and described constant vigilance of, but also understanding and empathy towards, their them. It is possible that having an autistic brother or sister with complex needs may present challenges and needs for siblings that are distinct from those of siblings of autistic children with fewer complex needs or indeed other disabilities, such that these siblings are taking on the responsibility and strain of caring roles (e.g., Jones et al. 2020; Zucker et al. 2022). The siblings may therefore be developing the ability to compromise, and to cope, with being subject to different expectations in their own behaviours and the expectation of the level of support and care they are providing to their autistic brother or sister, resulting in less quality time with their parents.

### 4.1 Limitations

This study has some limitations, we interviewed each sibling only once and therefore did not form extended relationships with the children. However, in‐depth information was given from all the siblings during the interviews, familiarisation visits were conducted before the interviews to ensure siblings were as comfortable and confident as possible to share their views. The presence of a parent during two of the interviews provided a level of comfort for the siblings but may also have dissuaded the children from being completely open during the interview. However, it was felt that it was more important that the siblings were able to participate in the way they felt most confident. It should also be considered that whilst the interviewer was transparent about her parent carer status, this may have also had a negative impact on how much the siblings wanted to share.

Additionally, we also only sought to understand one half of the sibling dyad, the non‐autistic sibling, as the majority of the autistic children were either non‐verbal or had very limited verbal capacity. Furthermore, only parents reported on their autistic child's ‘complex care needs’. As individual family dynamics may have an impact on how ‘complex care’ is perceived, it was important to allow for flexibility on this point and this was a deliberate part of the study design. However, the concept of complex care needs in the current research may not fully overlap with the sub‐groupings found in other studies that have used quantitative measures to delineate subgroups based on behavioural complexity (e.g., Rixon et al. 2021; Rixon, Hastings, and Kovshoff 2022). Despite these limitations, the findings provide a strong foundation for the understanding of how autism and complex care needs affect a non‐autistic child in their sibling relationship with an autistic brother or sister.

### 4.2 Future Research

There is a need to continue exploring the relationships of the siblings of autistic children, with an emphasis on understanding how the varying complexities and individualities of each sibling family group affect each member of the family and each sub‐system relationship. For example, there is scope to further explore how the communication ability of the autistic individual may affect the sibling relationship. Future research could include using adult, or young adult, siblings to carry out the interviews, perhaps providing a more intimate interview. An increase in rapport may be gained through the mutual understanding both the interviewee and the interviewer would bring to the experience. Additionally, there is further scope to explore how other factors such as age, the severity of challenging behaviour and the complexity of care needs affect the sibling relationship and sibling outcmes.

Whilst there has been an increase in research exploring the dynamics of interpersonal relationships often from a family systems perspective (Cox 2010; Schmeer et al. 2021; Wright and Benigno 2019), there remain gaps in the understanding of how the siblings of autistic individuals with complex care needs need support both within the family home and in external environments. Emphasis should be placed on areas such as education and social activities and any future caring roles these children and young people may have.

## Ethics Statement

Full ethical approval was granted through the University of Warwick Humanities and Social Sciences Research Ethics Committee (approval number: HSSREC 96/21‐22).

## Consent

Written informed consent was obtained from the parents/guardians and verbal assent given by the participants.

## Conflicts of Interest

The authors declare no conflicts of interest.

## Data Availability

The study data will not be made available.

## Appendix A

### Interview Protocol

#### Interview Outline and Proposed Questions

The interview should be relaxed, informal and open enabling the siblings to express their thoughts and feelings about their relationships with their brother or sister.

#### Opening

Thank you for agreeing to take part and for meeting me today. As you know, I am interested in understanding the relationships between brothers / sisters (delete as appropriate) who have a brother / sister (delete as appropriate) with autism and who need a lot of care.

You can say whatever you want to me today, I'm going to ask you some questions but there are no right or wrong answers. This is all about you telling me about you and your brother/sister. You are one of about 12–15 people that I will be talking to over the next month and this is because everyone has a different experience.

I will be using this (show Dictaphone) to record this interview and then I will transcribe the recordings, that means I will write down everything you tell me today. They are anonymised so no one will be able to link the interview back to you. You can stop or withdraw at any time.

Do you have any questions?

#### Interview Outline

Questions may include:

1. Tell me about you and your family, who lives here?
2. How would you describe your brother/sister to someone who doesn't know them?
3. Tell me about living with X; What's it like being X's brother or sister.
  - Prompt:
    - What are the best bits?
    - Are there any difficult bits?
    - What helps you handle difficult bits? Who helps? Does that work?
4. How does your brother/sister behave towards you?/How do you get on?
  - Prompt:
    - How does it make you feel to be with your brother/sister?
5. Do you spend much time with your brother / sister?
  - Prompt:
    - What do you do together OR Why is that?
    - What else do you do together?
    - What do you do together with your parent(s)/other brothers and sisters?
    - What did you do together last weekend?
6. Do you spend lots of time with you friends?
  - Prompt:
    - Why do you think that is?
    - Do your friends come to your house?
    - Do you friends spend time with your brother/sister too?
7. Do you think things between you and your brother/sister are the same now as when you were younger, or are they different?
  - Prompt:
    - How are they different?
8. Is there anything you would want to be different about how things are between you and your brother/sister, or is everything okay as it is?
  - Prompt:
    - Do you and your brother/sister ever fight/quarrel?
    - Why do you think that is?
9. Is there anything else you'd like to tell me about you and your brother/sister?

Note that if diagnosis is raised by the child, could ask about: how they would explain diagnosis to others; what support they get/they would like.
